# Migration and Degradation in Composting Environment of Active Polylactic Acid Bilayer Nanocomposites Films: Combined Role of Umbelliferone, Lignin and Cellulose Nanostructures

**DOI:** 10.3390/polym13020282

**Published:** 2021-01-16

**Authors:** Magdalena L. Iglesias-Montes, Francesca Luzi, Franco Dominici, Luigi Torre, Liliana B. Manfredi, Viviana P. Cyras, Debora Puglia

**Affiliations:** 1 Instituto de Investigaciones en Ciencia y Tecnología de Materiales (INTEMA), Facultad de Ingeniería, Universidad Nacional de Mar del Plata-Consejo de Investigaciones Científicas y Técnicas (CONICET), Av. Colón 10850, 7600 Mar del Plata, Argentina; ml.iglesiasmontes@gmail.com (M.L.I.-M.); lbmanfre@fi.mdp.edu.ar (L.B.M.); vpcyras@fi.mdp.edu.ar (V.P.C.); 2 Civil and Environmental Engineering Department, UdR INSTM, University of Perugia, Strada di Pentima 4, 05100 Terni, Italy; francesca.luzi@unipg.it (F.L.); franco.dominici@unipg.it (F.D.); luigi.torre@unipg.it (L.T.)

**Keywords:** poly(lactic acid), cellulose nanocrystals, lignin nanoparticles, umbelliferone, active food packaging, disintegration in compost, overall migration

## Abstract

This study was dedicated to the functional characterization of innovative poly(lactic acid) (PLA)-based bilayer films containing lignocellulosic nanostructures (cellulose nanocrystals (CNCs) or lignin nanoparticles (LNPs)) and umbelliferone (UMB) as active ingredients (AIs), prepared to be used as active food packaging. Materials proved to have active properties associated with the antioxidant action of UMB and LNPs, as the combination of both ingredients in the bilayer formulations produced a positive synergic effect inducing the highest antioxidant capacity. The results of overall migration for the PLA bilayer systems combining CNCs or LNPs and UMB revealed that none of these samples exceeded the overall migration limit required by the current normative for food packaging materials in both non-polar and polar simulants. Finally, all the hydrophobic monolayer and bilayer films were completely disintegrated in composting conditions in less than 18 days of incubation, providing a good insight on the potential use of these materials for application as active and compostable food packaging.

## 1. Introduction

The growing attention to the concept of active packaging is mainly driven by the growing population and the increase of food products demand and consumer trends [1]. In this context, the preservation and the safety of food products represent a crucial focus, which claimed the interest of academic research and the attention of manufacturers at different levels. The strategic solution can be found in the development of active packaging solutions that contain additives able to extend or maintain the shelf-life and the quality of food products [2,3]. The development of active solutions is contributing vigorously to the reduction of spoilage, food waste, food recalls, and foodborne illness outbreaks. Additionally, the implementation of active packaging systems containing biobased and/or biodegradable polymers represents a sustainable and also ecofriendly response to the problem. At the same time, the introduction on active additives in multifunctional system helps to prevent the safety of human health condition. These active technologies are used not only to develop food applications, but they have relevance also for packaging of pharmaceuticals, cosmetics and other consumer goods products [4,5]. Packaging systems play a crucial role in the protection of goods from external contamination, deterioration, physical damage [6] and biological changes. In Europe, the production of traditional plastics reached 49 million of T/y, almost 40% of which is used for packaging purposes [7]. Nowadays, besides the traditional barrier and physic-mechanical resistance features, new functionalities are required to extend the expiration date or the shelf-life of the products. Safer packaged food can be addressed if these new materials, called “active packaging materials”, are used to absorb unwanted substances such as heavy metals, and to protect against UV, moisture and oxidation, while conventional packaging materials cannot actively control the reactions of moisture, light and oxygen with sensitive foods [8].

Essentially, two methodologies can be pursued to enhance and modulate the shelf-life of food products: Incorporation of active molecules into monolayered packaging-based systems or development of multilayer polymeric systems to modulate the exchange of gas flow between the external and internal parts of the packaging [9,10]. The development of active polymeric based materials is centered on the incorporation of antioxidant molecules into the packages and the migration to the food [11]. Antioxidant packaging seeks to stop or limit the oxidation of different food components, principally, proteins and lipids, which leads to the deterioration of organoleptic and physical properties. Polymeric systems, and specifically biodegradable polymers, are the preferential materials adopted in packaging and in food packaging application. Green systems represent an eco-friendly option that can reduce the amount of wastes in landfills respect to petroleum/traditional based polymers, minimizing the greenhouse gas emissions during the production and the end-of-life disposal [12]. At the same time, these materials generally show poorer barrier properties towards gases than traditional ones. Biodegradable systems can be good carrier substrates of active principles because of their adaptability to controlled release and, also, they can be combined through the production of polymeric blends or multilayer formulations [13].

As described previously by us [10], bilayer films represent a strategic alternative to improve/modulate the structural properties, here the physical and functional properties, of package systems. Thanks to the advantages of versatility, functionality and convenience, multilayer food packaging has gained significant interest. As a single entity, multilayer packaging combines the benefits of each monolayer in terms of enhanced barrier properties, mechanical integrity and functional properties. Of late, apart from conventional approaches such as coextrusion and lamination, nanotechnology have been also used in the preparation of composite multilayer films with improved physical, chemical and functional characteristics [14]. The proposed research activity is the development of poly(lactic acid) (PLA) bilayer nanocomposites films containing lignin and cellulose as active and structural reinforcement, in combination with a coumarin, umbelliferone. PLA is a thermoplastic linear aliphatic polyester, obtained from renewable resources [15], accepted by US Food and Drug Administration (FDA) as a food contact matter, non-toxic or carcinogenetic, transparent, easy to process and economically feasible, and it is utilized to produce packaging for short shelf-life uses [10]. Cellulose nanocrystals (CNCs) and lignin nanoparticles (LNPs) have been also used as nanoreinforcements to improve the properties of biobased polymeric systems, based on PLA and its blends, for the food packaging sector [16,17]. Lignin NPs provide UV stabilization and antioxidant properties [17,18], they can provide effective mechanical reinforcement [19], as well as UV protection and antibacterial capabilities. They are also a promising green solution useful against dangerous microorganisms and bacteria [20,21]. With a similar aim, cellulose nanocrystals, which are characterized by low density, high biocompatibility and stiffness [22], can contribute to modulate the overall thermomechanical properties of polymeric materials, due to the synergic interactions of crystal nucleation, tortuosity and chain immobilization [23,24]. Umbelliferone (7-hydroxycoumarin) (UMB), an active ingredient, is a natural phenolic extract extensively spread in plants of the coumarin family with antioxidant and antiseptic effects [25,26].

PLA monolayer films containing CNCs or LNPs and UMB were extruded and successfully layered by thermo-compression and investigated to analyze the fundamental properties for the final application in the packaging sector, as proposed by Iglesias and co-authors [10] in a previous paper. Here, we focused the attention on the effect of cellulose nanocrystals, lignin nanoparticles and umbelliferone in monolayer or bilayer films towards functional and compostable properties of poly(lactic acid)-based films developed for the packaging sector. The disintegration study in composting condition was considered to simulate the final end of life of processed systems and to evaluate the influence of different components.

## 2. Materials and Methods

### 2.1. Materials

**Figure 1:**
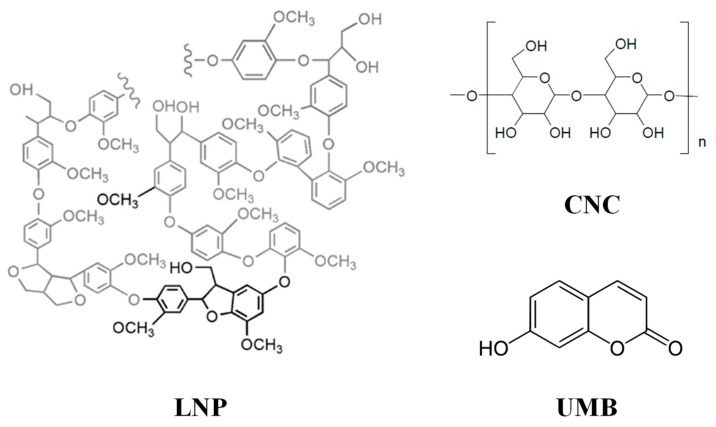
Chemical structures of lignin (LNP), cellulose (CNC) and umbelliferone (UMB).

Poly(lactic acid) (PLA 3051D, M_n_ = 14,200 g mol^−1^, specific gravity = 1.25 g cm^−3^, melt flow index (MFI) = 7.75 g 10 min^−1^ (210 °C, 2.16 kg)) was purchased from Nature Works^®^ (Minnetonka, MN, USA). Pristine lignin was supplied by CRB (Centro Ricerca Biomasse, University of Perugia, Perugia, Italy) and lignin nanoparticles (LNPs) were synthesized by hydrochloric acidolysis as previously reported by Yang et al. [20]. Microcrystalline cellulose (MCC, dimensions of 10–15 µm), utilized as cellulose nanocrystals precursor during the hydrolysis process, was purchased from Merck Life Science (Milano, Italy). Cellulose nanocrystals (CNCs) were synthesized as previously reported [27]. Umbelliferone (UMB) (C_9_H_6_O_3_, 7-Hydroxycoumarin with 98.5% of purity, average M_w_ = 162.14 kg mol^−1^) and all the chemical reagents were provided by Sigma-Aldrich^®^ (Milan, Italy) and used as received. The chemical structures of the lignocellulosic nanostructures and UMB are shown in Figure 1.

### 2.2. Film Preparation

Four different PLA-based monolayer films were obtained by melt blending in a twin-screw microextruder (DSM Xplore 15 Micro Compounder, Xplore Instruments BV, Sittard, The Netherlands): neat PLA, two PLA nanocomposites with 3 wt% of CNCs or LNPs, and PLA active film with 15 wt% of UMB. CNCs, LNPs and UMB have been selected according to previous results obtained by the authors on separated or combined use of CNCs and LNPs in poly(lactic acid) [28], while the amount of UMB was selected on the basis of dispersibility results and its effect of transparency and antibacterial response when introduced in polymeric film formulations [25]. All based materials were dried prior processing in an air circulating oven at 50 °C overnight in order to eliminate moisture traces. The processing parameters were tuned for each film formulation. Screw speed was set at 100 rpm over 3 min, using a temperature profile of 180–190–200 °C through the three different extruder heating zones. CNCs, LNPs and UMB were added after 2 min of PLA mixing procedure to elude their degradation. Monolayer films with 50 mm of width and nominal thickness around 80–85 µm were obtained by using the adequate nozzle. Subsequently, the bilayer films were obtained by means of compression in a hot press. Different pairs of PLA-based monolayer films were combined and compressed at 155 °C for 1 min at a pressure of 25 bar followed by a cooling cycle. Six kinds of bilayer films were obtained. The prepared material formulations are shown in Table 1.

### 2.3. Wettability

The surface wettability of films was analyzed by measuring the water contact angle (WCA) values using the Sessile Drop method. The contact angle measurements were studied by using deionized water (volume 20 μL) conducted by FTA 1000 Analyser at room conditions (FTA1000, First Ten Angstroms, Inc. Portsmouth, UK) equipped with a camera and Drop Shape Analysis SW21; FTA32 2.0 software (First Ten Angstroms, Inc., Portsmouth, UK). Drops of distilled water were placed on the material surfaces and the side profiles of drops were analyzed with the DROP image Software. The average value of the contact angles of five drops randomly placed onto the film surfaces was reported. WCA corresponding to bilayer films were measured on both sides of the films.

### 2.4. Overall Migration Test

The overall migration (OM) tests were performed in triplicate by using ethanol 10% (*v/v*) as aqueous food simulant (simulant A) (EC 2011) [29] and isooctane (simulant D) as the alternative fatty food simulant (EC 2002) [30]. Rectangular strips of 10 cm^2^ were completely immersed into 10 mL of each food simulant [29]. Samples were maintained in an oven at 40 °C for 10 days in ethanol 10% (*v/v*) and in a climate chamber at 20 °C for 2 days in isooctane, according to EN-1186 standard [30]. After the incubation time, samples were removed and the simulants were totally evaporated at 105 °C. The non-volatile residues were weighed using an analytical balance (±0.1 mg) and the overall migration values, expressed in mg kg^−1^ of simulant, were calculated and recorded.

### 2.5. Disintegration in Composting Conditions

Disintegrability in composting conditions was performed following the ISO-20200 standard. The method determines the degree of disintegration of plastic materials under simulated aerobic composting conditions in a laboratory-scale test [31]. In order to prepare the solid synthetic waste, a specific quantity of compost inoculum, supplied by Gesenu S.p.a. (Perugia, Italy), was mixed with sawdust, rabbit food, starch, sugar, oil and urea, maintaining substrate water content around 50%. Aerobic conditions during the test were guaranteed using perforated boxes and softly hand mixing the solid soil every day. The holes in the boxes provide gas exchange between the inner atmosphere and the outside environment. PLA neat and nanocomposites monolayer and bilayer films (surface: 25 mm × 25 mm) were weighed and buried into the organic substrate at 4–6 cm depth and incubated at 58 °C and 50% of humidity. Film samples were previously included into meshed plastic bags to let the films contact freely with the soil as not to lose small material fragments after a long-time exposure. The tested samples were then recovered at different times (1, 4, 8, 11, 14 and 18 days), washed with deionized water, dried in oven at 37 °C for 24 h, and weighed in an analytical balance (±0.1 mg). Photographs of the samples were taken for visual comparison. The degree of disintegration (D) of every sample at each time period was calculated by using Equation (1):

(1) $$D\;=\;\left[\frac{\left(m_{i}-m_{r}\right)}{m_{i}}\times 100\right],$$

where *m_i_* and *m_r_* are the initial and residual dry weights of samples, respectively. According to the European Standard, the materials tested can be considered disintegrable when 90% of the plastic sample weight is lost within 90 days of analysis.

### 2.6. Surface Characterization

The morphological aspect/modifications after composting tests were examined by means of field emission scanning electron microscopy (FESEM, Supra 25-Zeiss, Oberkochen, Germany) after gold sputtering and by using an accelerating voltage of 5 kV.

### 2.7. Statistical Analysis

Results expressed as mean ± standard deviation were analyzed by one-way analysis of variance (ANOVA). Means were tested with the Tukey’s test for paired comparison, with a significance level α = 0.05, using OriginPro 8 software (Northampton, MA, USA). Average values followed by different letters have statistically significant difference (*p* ≤ 0.05).

## 3. Results

In the present work, functional properties of active PLA-based mono and bilayer films and their suitability for biodegradable food packaging applications were assessed by analyzing their behavior under food simulants and composting conditions during several days. However, before examining in detail these crucial physicochemical properties of the novel films, it is interesting to describe some other basic properties of the materials. A fully characterization of the films has been carried out in our previous study [10] and their most relevant thermal and antioxidant properties, with regards to the current investigation, are summarized in Table 2. Briefly, the incorporation of CNCs and LNPs nanoparticles into the PLA matrix of monolayer films did not significantly modify its glass transition (*T*_g_) and melting (*T*_m_) temperatures, while the addition of the microscale active ingredient (UMB) caused a reduction in *T*_g_, *T*_m_ and *T*_c_ and a consequent increment in the PLA crystallinity percentage (χ_c_), attributed to a plasticizing effect of umbelliferone [32]. Similar reductions in *T*_g_ were observed for the bilayer systems containing the active ingredients (AIs). In general, the crystallinity capability of PLA was enhanced in the multilayer films probably due to a reorganization of the crystalline phase during the thermo-compression molded films assembled process. This was evidenced by reductions in *T*_c_ values (118 and 99 °C for PLA and PLA/PLA, respectively; 116 and 108 °C for PLA_3CNC and PLA/PLA_3CNC, respectively; 126 and 112 °C for PLA_3LNP and PLA/PLA_3LNP, respectively).

PLA-nanocomposites and active monolayer films have proved to be more thermally stable than neat PLA, according to their higher maximum degradation temperature (*T*_d_) under nitrogen atmosphere. In general, bilayer films exhibited increased thermal stability compared to the corresponding monolayer films attributed to the increment in the crystallinity percentage [10]. However, PLA/PLA_15UMB presented the lowest heat resistance probably due to a plasticizing effect of UMB and a diminution in the χ_c_ in the bilayer system. Regarding the antioxidant capacity of the mono and bilayer films, LNPs produced the highest antioxidant activity in the monolayer formulations, while the combined incorporation of LNPs and UMB in the PLA bilayer films induced the highest antioxidant activity due to the synergistic effect of both additives [25,33].

### 3.1. Wettability

Knowledge of the surface properties of polymers is useful when designing a packaging material for a specific application [34]. Surface hydrophobicity is an important issue for materials intended to be in direct contact with foodstuff, especially when used for high moisture foods as well as when submitted to high humidity conditions during storage and/or transport [35]. The wettability of the formulations was investigated by measuring the water contact angle (WCA) and the corresponding values are reported in Table 3. Neat PLA monolayer film showed a WCA of (72.3 ± 1.6)°, which is consistent with those found in literature [27,36]. The addition of nanoadditives induced significant variations (*p* ≤ 0.05, Table 3) of PLA wettability compared to the neat control. PLA_3CNC presented a reduction of around 11% of the contact angle (64.4 ± 0.9)°, showing a significant decrease in the hydrophobic character of the PLA matrix surface, probably due to the incorporation of hydroxyl groups on the surface of the film [37]. On the other hand, the presence of LNPs improved the surface water resistance of PLA_3LNP, increasing the WCA value in around 8% (79.1 ± 2.0)°. Lignin molecules are composed of an aromatic skeleton and non-polar hydrocarbon chains which grant its hydrophobic character [36,38]. Regarding the active ingredient, it was observed that, although umbelliferone is a hydrophobic coumarin compound [39], its introduction into the PLA_15UMB film did not significantly (*p* > 0.05) affect the wettability properties of the polymer matrix, showing a value of (73.2 ± 1.8)°. It is known that the wettability of the polymer matrix is strongly related to the surface chemical and topographical properties of the film [40]. Then, it could be possible that the UMB was not exposed on the monolayer film surface and consequently did not affect the wettability of PLA_15UMB. Similar results were obtained with PLA/CNC nanocomposite films [27].

PLA/PLA bilayer film presented a significantly higher WCA value (*p* ≤ 0.05) than that of the monolayer neat control, producing a decrease in the wettability of the sample surface. This result could be related to the almost six-fold higher degree of crystallinity of the multilayer system reached during the thermo-compression (Table 2) [41]. Similar results were reported for nanocomposite bilayers based on chitosan and PVA, where the increase in the hydrophobicity of the material was attributed to matrix transformations during bilayer processing at temperature [42]. In the case of materials made by different formulation films assembly, measurements on both sides of the bilayer samples were performed. No statistically significant differences in the contact angles were found between the two sides of PLA/PLA_3CNC bilayer film and with PLA monolayer sample (*p* > 0.05) (i.e., it was not registered a significant reduction in WCA as with PLA_3CNC monolayer film). The lower content of CNCs hydrophilic filler, the higher degree of crystallinity of the matrix (Table 2) and a possible diffusion of the nano additive into the bulk of the matrix during the thermo-compression process could be some explaining factors. In a similar way, wettability of all sample’s sides of PLA/PLA_3LNP, PLA_3LNP/PLA_15UMB and PLA were alike (*p* > 0.05), being slightly more hydrophobic the films surfaces containing LNPs [43,44]. On the other hand, the combination of hydrophilic CNCs and hydrophobic UMB in the bilayer system (PLA_3CNC/PLA_15UMB) induced a significant difference in WCA between both sides (*p* ≤ 0.05).

Surprisingly, the PLA/PLA_15UMB showed a WCA around 16° higher than that of PLA homopolymer and PLA_15UMB monolayer (*p* ≤ 0.05), obtaining a significant improvement in the hydrophobic character of PLA. This result could be related to the fact that the active ingredient produces a plasticizing effect promoting the flow of PLA layers and of UMB itself during the thermo-compression process, exposing the hydrophobic AI on both film side surfaces. Moreover, the PLA/PLA_15UMB formulation presented the lowest film thickness (Table 2) among the multifunctional bilayer films, in agreement with the assumption of a high content of UMB superficially exposed. Finally, a contact angle higher than 65° is typical of hydrophobic surfaces [38,39] and, in consequence, all formulated films turned out to be water resistant (only the monolayer PLA_3CNC sample showed an angle around 65°).

### 3.2. Overall Migration Test

Overall migration of non-volatile substances from food packaging materials into foodstuff is one of the main important properties of polymeric devices that are requested to be in compliance with current regulations [45]. The test was performed using two different food simulants in order to analyze the polymeric films behavior upon two different types of foodstuffs. Ethanol 10% (*v/v*) (simulant A) was chosen as the aqueous food type simulant and isooctane (simulant D) as the alternative simulant for fatty food performance. The overall migration limit (OML), set up by the current legislative Council of the EU [30], is the maximum permitted total mass of substances that can migrate into the food simulant and should not exceed a value of 60 mg per kg food (mg kg^−1^). Test results for neat and nanocomposites PLA monolayer and bilayer films are summarized in Table 4.

In general, the overall migration levels using simulant A for PLA-based formulations were within the migration permitted limits, registering a migration level of (18.6 ± 2.0) mg kg^−1^ for neat PLA film and no significant differences (*p* > 0.05) with the migration levels of monolayer formulations PLA_3CNC and PLA_3LNP, and bilayer formulations PLA/PLA, PLA/PLA_3CNC and PLA/PLA_3LNP. However, as expected based on previous work reports [23], overall migration values of PLA formulation films incorporated only with the AIs (i.e., PLA_15UMB and PLA/PLA_15UMB) were above the OML (*p* ≤ 0.05). Moreover, it was known that UMB has a slight solubility in hot water and a high solubility in ethanol [44]. The further incorporation of nano-additives (CNCs or LNPs) into the active bilayer system, significantly enhanced (*p* ≤ 0.05) the migration performance of PLA_3CNC/PLA_15UMB and PLA_3LNP/PLA_15UMB, reaching levels below the OML and in compliance with the EU legislation. This outcome highlights the effectiveness of using multilayer systems combined with nanoparticles in order to retain the active agent in the PLA matrix, thus reducing diffusion through film thickness [46]. Migration values were lower for simulant D and, in this case, the overall migration levels for all PLA based formulations were below the migration permitted limits (60 mg kg^−1^ of simulant). Specifically, neat PLA showed the lowest migration level around (9.0 ± 0.4) mg kg^−1^. In general, the migration level increased incorporating AI in the polymer matrix. Unexpectedly, the combination of UMB and CNCs or LNPs in bilayer systems induced a slightly increased (*p* ≤ 0.05) migration.

At last, both formulations that were demonstrated to produce the highest antioxidant activities (i.e., PLA_3LNP and PLA_3LNP/PLA_15UMB; Table 2) were proven to be in perfect compliance with the EU Regulation regarding the overall migration from plastic materials and articles intended to come into contact with food

### 3.3. Disintegrability in Composting Conditions

**Figure 2:**
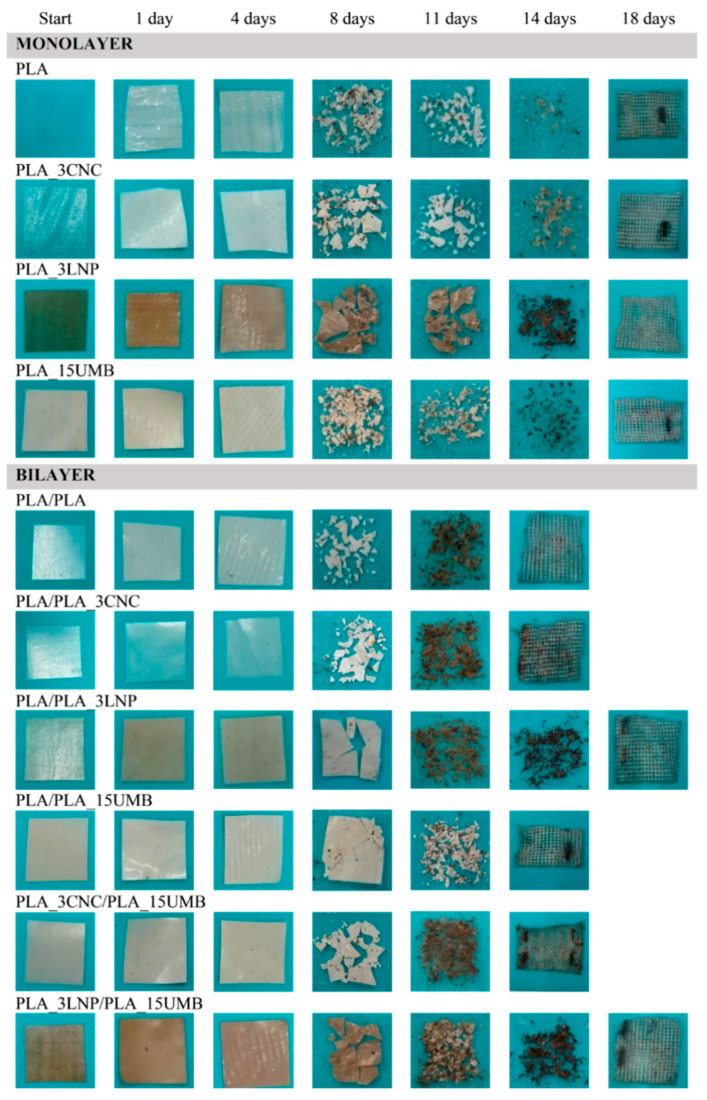
Visual observation of PLA (neat and nanocomposites) monolayer and bilayer films at different stages of incubation in composting conditions.

Currently, plastic packaging waste is one of the biggest pollutants of the environment worldwide, thus the development of new food packaging materials with biodegradable or compostable properties is of great importance in the attempt to reduce the ecological impact [47,48]. In order to assess the compostable character of the manufactured green-based polymeric systems, the disintegration of the PLA-based mono and bilayer films was carried out under composting conditions. Composting is the natural process of decomposing organic solid matter, which is mainly performed by microorganisms such as bacteria, fungi, and actinomycetes [49]. It is well known that PLA degradation in compost usually involves four main phenomena, specifically, water absorption, hydrolysis of the polymer chains causing ester cleavage and formation of lactic acid, lactide and oligomers [50], solubilization of the low molecular weight compounds, and finally diffusion and metabolization of the soluble compounds by the microorganisms [51,52]. Therefore, the factors that accelerate the hydrolysis tendency of PLA ultimately control the degradation of PLA, namely, temperature and humidity level, among others [53]. It was reported that, in composting environments of high humidity and temperature (55–70 °C), PLA polymers are found to degrade rapidly [54,55]. Accordingly, in the present study, the PLA test samples were buried in composting soil, keeping constant the soil temperature and moisture content at 58 °C and 50%, respectively, in agreement with the ISO-20200 Standard. The preliminary phase of the results analysis was the visual examination of all the degraded samples after contact with compost at different incubation times by means of comparing photographs taken for this purpose (Figure 2). Samples were monitored over 18 days and pictures of the empty meshed bags are included in Figure 2 to show the total disintegration of materials. All samples exhibited a considerable surface deformation, loss of transparency and whitening just after the first day of incubation. This outcome could be indicating that water uptake and hydrolytic degradation of the polymer matrix have started and, in consequence, have induced a change in the refraction index of the films [56,57]. This phenomenon is very common for PLA-based materials and many authors have described the same behavior [58,59,60].

The color of the different samples changed and these variations are related to the alteration in the polymer crystallinity during the degradation process [61]. The amorphous chains of the polymer structure are the ones preferentially hydrolyzed and degraded, thus changing the ratio of crystalline to amorphous domains in PLA. Moreover, a further crystallization of the PLA matrix could occur due to a possible increase in chain mobility induced by the effect of the test temperature (58 °C), which is very close to the glass transition temperature (~60 °C) of PLA [36,61]. These factors lead to the embrittlement of the samples [17]. In fact, after eight days of incubation films became breakable and after 11 days only small fragments could be recovered from the compost soil.

**Figure 3:**
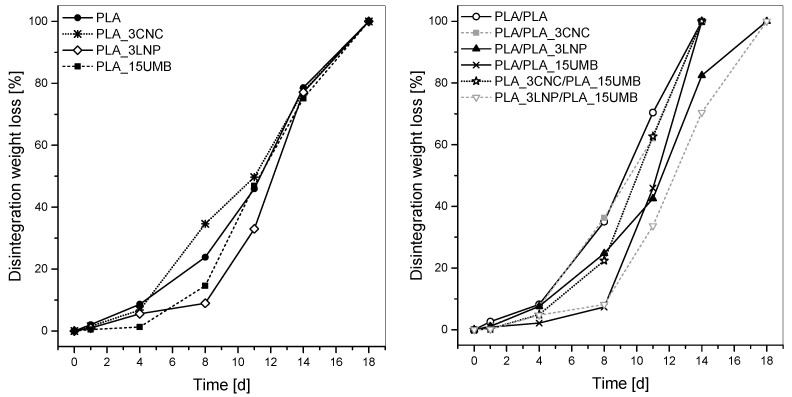
Disintegrability percentage values of PLA (neat and nanocomposites) monolayer (**left**) and bilayer (**right**) films at different stages of incubation in composting conditions.

The disintegrability was also evaluated in terms of mass loss at different incubation times (Figure 3). Regarding the monolayer films (Figure 3, left), it was observed that after 18 days all four formulations reached 100% of disintegration, but with slightly different degradation rates during the process. The degradation behavior of nanocomposites strongly depends on the hydrophilicity/hydrophobicity and the dispersion of the nanofillers [37]. Over the first four days of incubation, disintegrability values remained low and constant for all systems, but considerably increased after eight days when films became breakable and discrepancies in mass loss between materials could be seen. PLA_3CNC presented the highest mass loss among them (around 35%), likely due to the presence of hydroxyl groups belonging to the nanocrystalline structure that plays a catalytic role on hydrolysis of the ester groups of the PLA, in agreement with several investigations about PLA-CNC nanocomposite degradation [62,63,64]. On the other hand, PLA_3LNP (mass loss around 9%) and PLA_15UMB (mass loss around 15%) presented lower disintegration values than pure PLA (around 24%). This behavior could be attributed to the hydrophobic character of the fillers [37], and to the fact that both ingredients have chemical characteristics with great resistance to microorganism’s attack and to the penetration of destructive enzymes into the cell wall [65,66,67,68,69]. All these features make their degradation more difficult.

Bilayer films (Figure 3, right) followed a similar tendency than the monolayer materials towards disintegrability in compost. However, formulations containing CNCs or UMB or combination of both (i.e., PLA/PLA, PLA/PLA_3CNC, PLA/PLA_15UMB and PLA_3CNC/PLA_15UMB) degraded faster than the rest of materials, disintegrating completely after 14 days. This could be explained by the possible presence of pores generated during the thermo-compression assembly of bilayer films, which would favor the process of disintegrability in composting since the internal structure is more easily accessible by water and microorganisms. PLA/PLA_3LNP and PLA_3LNP/PLA_15UMB bilayer films reached 100% disintegrability on day 18, highlighting the effect of LNPs in delaying the degradation rate of PLA. In general, all the results are consistent with the results in term of water contact angle measurements (i.e., the higher the wettability of the film surface the faster the disintegration rate; Table 3). This was particularly accurate for disintegration behavior after eight days in composting soil.

**Figure 4:**
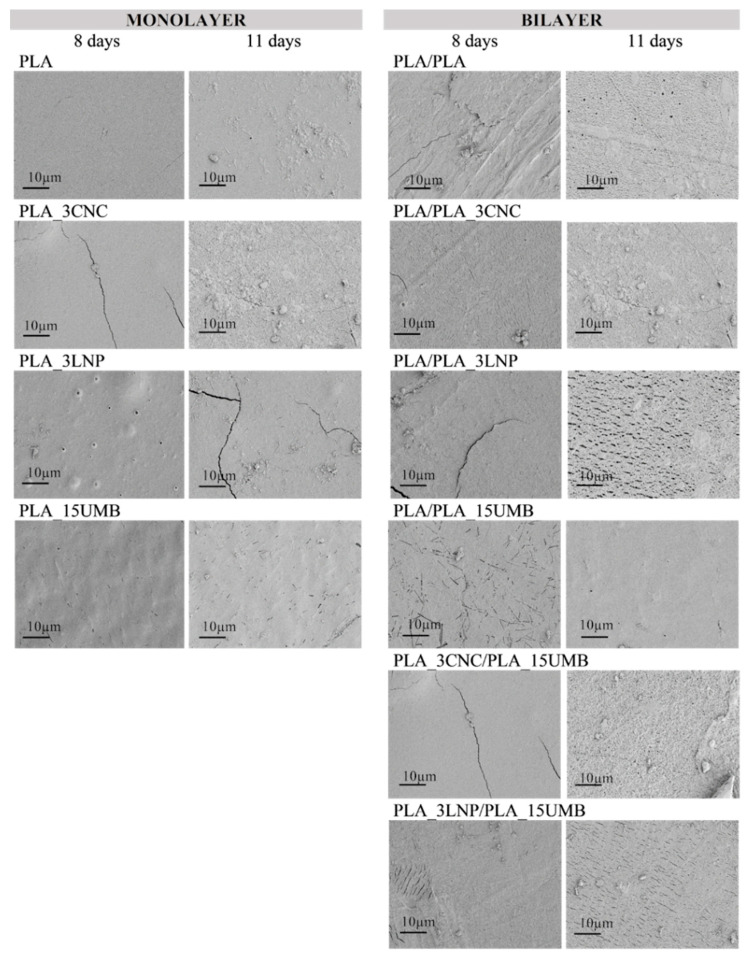
FESEM images of PLA (neat and nanocomposites) monolayer and bilayer film surfaces after eight and 11 days of incubation in composting conditions.

Surface morphological characterization of PLA-based systems after different incubation times was also carried out. Figure 4 shows the FESEM images of neat and nanocomposites PLA monolayer and bilayer films after eight and 11 days in composting conditions. Clear surface erosion with the appearance of holes and porous structures on PLA and all PLA-based formulations was observed. Moreover, the materials’ surface was characterized by regular transverse striations, especially noticeable for PLA/PLA, PLA/PLA_3LNP, PLA_3CNC/PLA_15UMB and PLA_3LNP/PLA_15UMB bilayer films. Similar results were evidenced by Yang and co-authors [36] while studying the degradation of PLA-LNP bionanocomposites. The authors associated the presence of these deeper grooves to a more aggressive erosion of the amorphous macromolecules on the film surface. The complete disintegration of the films in composting conditions is a promising outcome that highlights the sustainable post-use alternative for the obtained materials.

## 4. Conclusions

Fully biobased bilayer films of PLA containing lignocellulosic nanostructures and active ingredient were successfully produced through a green solvent-free simple process, including monolayers extrusion and thermo-compression assembly. The development of multilayer polymeric systems was used as a strategy to elaborate active packaging solutions in order to enhance and modulate the shelf-life of food products. A number of functional properties of the produced PLA-based systems have been characterized. The combined incorporation of lignin nanoparticles and umbelliferone in the bilayer PLA_3LNP/PLA_15UMB film induced the highest antioxidant capacity as a result of the synergistic effect of both additives. All the designed materials showed hydrophobic character, which is an important property for materials intended to be used as food packaging. PLA_3LNP, PLA/PLA_15UMB and PLA_3LNP/PLA_15UMB films presented the lowest wettability due to the presence of non-polar LNPs and UMB particles. It was also evidenced that the wettability of the polymer surface is strongly related to its chemical and topographical properties.

Regarding the overall migration in polar and non-polar food simulants, most of the samples were in compliance with the EU legislation for plastic materials intended to come into contact with foodstuff, except for PLA_15UMB and PLA/PLA_15UMB formulations that exceeded the overall migration limits in ethanol 10% *v/v*. However, the assembly of PLA_15UMB with PLA_3CNC or PLA_3LNP monolayers produced an efficient barrier effect due to the well dispersion of the nanofillers enhancing the overall migration performance of both bilayer films. Disintegration in composting conditions was successfully accomplished within just 18 days of incubation in a laboratory scale test, showing that a sustainable end-life option is possible for the developed materials.

Finally, the implementation of these antioxidant packaging systems containing biobased and biodegradable polymers could represent a sustainable and also ecofriendly solution for film applications, such as food packaging.

## Figures and Tables

**Table 1:** Name and description of material formulations.

Film Formulation	Name
**MONOLAYER**	
Pure PLA	PLA
PLA + 3% CNCs	PLA_3CNC
PLA + 3% LNPs	PLA_3LNP
PLA + 15% UMB	PLA_15UMB
**BILAYER**	
Pure PLA//Pure PLA	PLA/PLA
Pure PLA//PLA + 3% CNCs	PLA/PLA_3CNC
Pure PLA//PLA + 3% LNPs	PLA/PLA_3LNP
Pure PLA//PLA + 15% UMB	PLA/PLA_15UMB
PLA + 3% CNCs//PLA + 15% UMB	PLA_3CNC/PLA_15UMB
PLA + 3% LNPs//PLA + 15% UMB	PLA_3LNP/PLA_15UMB

PLA (polylactic acid), CNC (cellulose nanocrystals), LNP (lignin nanoparticles), UMB (umbelliferone).

**Table 2:** Thermal and antioxidant characterization data of PLA (neat and nanocomposites) monolayer and bilayer films, including glass-transition temperature (*T*_g_), crystallization temperature (*T*_c_), melting temperature (*T*_m_), degree of crystallinity (χ_c_), thermal degradation temperatures (*T*_0_ and *T*_d_) and DPPH radical scavenging activity (RSA) (time = 1 h).

			Thermal Properties					Antioxidant Activity
	Thickness [µm]	^a^*T*_g_ [°C]	^a^*T*_c_ [°C]	^a^*T*_m_ [°C]	^a^*χ*_c_ [%]	^b^*T*_0_ [°C]	^b^*T*_d_ [°C]	RSA [%]
**MONOLAYER**								
PLA	54	63	118	150	5	284	312	-
PLA_3CNC	119	62	116	149	2	297	329	2.5
PLA_3LNP	140	63	126	152	3	306	350	80.4
PLA_15UMB	62	55	84	147	12	262	344	45.2
**BILAYER**								
PLA/PLA	82	60	99	153	28	316	352	-
PLA/PLA_3CNC	127	60	108	158	12	316	354	6.8
PLA/PLA_3LNP	119	60	112	152	9	290	317	62.0
PLA/PLA_15UMB	89	57	119	147	6	257	293	54.0
PLA_3CNC/PLA_15UMB	124	53	103	150	14	292	341	32.3
PLA_3LNP/PLA_15UMB	102	55	120	147	7	287	332	70.7

PLA (polylactic acid), CNC (cellulose nanocrystals), LNP (lignin nanoparticles), UMB (umbelliferone). ^a^ By DSC (Differential Scanning Calorimetry) I scan, ^b^ By TGA (Thermogravimetric Analysis).

**Table 3:** Water contact angle (WCA) values of PLA (neat and nanocomposites) monolayer and bilayer films.

		WCA [°]		WCA [°]
**MONOLAYER**				
PLA		72.3 ± 1.6 ^af^		
PLA_3CNC		64.4 ± 0.9 ^bf^		
PLA_3LNP		79.1 ± 2.0 ^cd^		
PLA_15UMB		73.2 ± 1.8 ^ad^		
**BILAYER**				
PLA/PLA	PLA side	77.6 ± 0.9 ^d^		
PLA/PLA_3CNC	PLA side	71.7 ± 1.1 ^af^	PLA_3CNC side	70.0 ± 0.8 ^af^
PLA/PLA_3LNP	PLA side	70.3 ± 1.0 ^af^	PLA_3LNP side	74.0 ± 2.0 ^ad^
PLA/PLA_15UMB	PLA side	88.2 ± 3.1 ^e^	PLA_15UMB side	88.9 ± 1.9 ^e^
PLA_3CNC/PLA_15UMB	PLA_3CNC side	67.9 ± 0.7 ^f^	PLA_15UMB side	73.3 ± 0.6 ^ad^
PLA_3LNP/PLA_15UMB	PLA_3LNP side	74.2 ± 2.4 ^ad^	PLA_15UMB side	72.1 ± 1.4 ^af^

^a–f^ Different superscript letters within the table indicate significant differences among formulations (*p* ≤ 0.05) according to the Tukey test.

**Table 4:** Overall migration characterization data of PLA (neat and nanocomposites) monolayer and bilayer films.

	Overall Migration [mg kg^−1^]	
	Ethanol 10% (*v/v*) 10 Days @ 40 °C	Isooctane 2 Days @ 20 °C
PLA	18.6 ± 2.0 ^a^	9.0 ± 0.4 ^a^
PLA_3CNC	17.2 ± 3.5 ^a^	11.0 ± 0.8 ^ab^
PLA_3LNP	18.4 ± 2.9 ^a^	10.7 ± 1.2 ^ab^
PLA_15UMB	81.3 ± 8.1 ^b^	12.8 ± 0.8 ^b^
PLA/PLA	18.4 ± 1.7 ^a^	10.7 ± 0.4 ^ab^
PLA/PLA_3CNC	21.7 ± 1.8 ^a^	11.3 ± 0.4 ^ab^
PLA/PLA_3LNP	21.2 ± 2.3 ^a^	10.7 ± 0.4 ^ab^
PLA/PLA_15UMB	76.8 ± 6.9 ^b^	12.2 ± 0.8 ^ab^
PLA_3CNC/PLA_15UMB	38.4 ± 3.5 ^c^	17.7 ± 2.9 ^c^
PLA_3LNP/PLA_15UMB	36.8 ± 5.8 ^c^	18.3 ± 1.2 ^c^

^a–c^ Different superscript letters within the same column indicate significant differences among formulations (*p* ≤ 0.05) according to the Tukey test.

## Data Availability

The data presented in this study are available on request from the corresponding author.
